# An impact of dietary intervention on blood pressures among diabetic and/or hypertensive patients with high cardiovascular disorders risk in northern Thailand by cluster randomized trial

**DOI:** 10.1002/jgf2.379

**Published:** 2020-09-22

**Authors:** Hirohide Yokokawa, Motoyuki Yuasa, Supalert Nedsuwan, Saiyud Moolphate, Hiroshi Fukuda, Tsutomu Kitajima, Kazuo Minematsu, Susumu Tanimura, Eiji Marui

**Affiliations:** ^1^ Department of General Medicine Juntendo University School of Medicine Tokyo Japan; ^2^ Department of Public Health Juntendo University School of Medicine Tokyo Japan; ^3^ Department of Social and Preventive Medicine Chiang Rai Regional Hospital Chiang Rai Thailand; ^4^ Department of Public Health Chiang Mai Rajabhat University Chiangmai Thailand; ^5^ Faculty of Social Science Kyorin University Graduate School of International Corporation Studies Hachioji Japan; ^6^ Department of School Health Faculty of Education Nagasaki University Nagasaki Japan; ^7^ Department of Public Health Nursing Mie University Graduate School of Medicine Tsu Japan; ^8^ Department of Human Arts Sciences University of Human Arts and Sciences Saitama Japan

**Keywords:** awareness, dietary, hypertension, intervention, nutrition, salt intake

## Abstract

**Background:**

Global sodium intake remains above the recommended levels to control blood pressure (BP). We aimed to evaluate the efficacy of a dietary intervention on BP through salt reduction among community‐dwelling participants with high risk of cardiovascular disorders (CVD).

**Methods:**

This cluster randomized trial (February 2012 to January 2013) included cooking instruction using the pocket salt meter among patients with diabetes and/or hypertension who were treated at health center in Thailand. Based on health centers, 8 clusters of eligible participants were randomly allocated to the 4 intervention and 4 control groups. Dietary intervention was performed at baseline, 1 month, and 3 months in intervention group. In both groups, systolic and diastolic BPs, and estimated 24 hours salt intake based on overnight urine samples were measured at baseline, 6 months, and 12 months.

**Results:**

A total of 753 participants were enrolled (374 in the intervention group and 379 in the control group). In the mixed‐effects model, there were significant difference in SBP and estimated salt intake after adjusting covariates at 6 months (adjusted differences between groups [95% CI]; −7.55 [−5.61 to −9.49] mm Hg *P* < .01; −0.66 [−0.40 to −0.92] g/day *P* = .03). However, these differences were not observed at 12 months (adjusted differences between groups [95% CI]; −1.83 [0.34 to −4.00] mm Hg *P* = .48; −0.42 [−0.17 to −0.67] g/day *P* = .16). There were no differences in DBP in both follow‐ups.

**Conclusions:**

These results may suggest the effectiveness of a visually based dietary intervention targeting salt intake reduction in short term, but the effectiveness discontinued in long term.

Clinical trial number: The International Standard Randomized Controlled Trial Number Register (ISRCTN39416277) on January 3, 2012.

## Graphical abstract

Our cluster randomized trial data revealed the effectiveness of a dietary intervention combining cooking instruction with visualization of estimated salt intake in reducing salt intake. This type of dietary intervention targeting salt intake reduction may contribute to BP management.

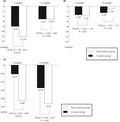

## 1 BACKGROUND

Hypertension is a major cause of cardiovascular events, both in developed and in developing countries. An estimated 1.39 (1.34‐1.44) billion people had hypertension in 2010, 349 (337‐361) million in high‐income countries and 1.04 (0.99‐1.09) billion in low‐ and middle‐income countries.^1^ Elevated blood pressure (BP) is estimated to cause 7.5 million deaths, about 12.8% of all deaths. Hypertension accounts for 57 million disability‐adjusted life years (DALYS) or 3.7% of total DALYS.^2^ High salt intake has been associated with high BP, thereby contributing to cardiovascular disease (CVD).^3^, ^4^, ^5^, ^6^ Salt intake reduction has been established as an important public health strategy for prevention of hypertension.^7^, ^8^, ^9^, ^10^ Hypertension (raised blood pressure ≥140/90 mm Hg) is common among Thais as well as worldwide and is on the rise. One out of 4 adult Thais has hypertension. Hypertension causes more than 50 000 deaths annually.^11^

Several intervention trials have shown that a low‐sodium diet has the potential to reduce BP.^12^, ^13^ In spite of these evidences, global sodium intake still does not reach the levels recommended by the World Health Organization (WHO) which is below 5 g/day, and Thailand is relatively higher sodium intake levels (5.31 g/day) in the world.^14^, ^15^, ^16^ Although the importance of salt reduction is emphasized in Thailand as well as worldwide, the evidences regarding salt reduction intervention are still limited and there are few trials to use visualization tools to inform participants of the salt content. Under this background, we conducted a cluster randomized trial (CRT) in the northernmost province of Thailand (Risk Patients by Advanced Health Education Intervention [RESIP‐CVD Study]).^17^ In the trial, the effect of an intensive health education intervention that used visualization tools to help increase awareness of daily salt intake was examined and baseline data were already reported.^18^ We aimed to evaluate the effectiveness of the intervention on blood pressure through salt intake reduction among those with a high CVD risk in northern Thailand using longitudinal data analysis.

## 2 METHODS

### 2.1 Study design

This study was a CRT examining the effect of an intensive health education intervention to reduce dietary salt intake among cardiovascular risk patients. The intervention consists of cooking instruction alongside informing estimated daily salt intake through overnight urinary sodium measurement and participants' visualization of salt level in their homemade food. The study was conducted from February 2012 to January 2013, and the baseline survey was conducted from February 17 to 30, 2012. Detail of the study protocol was already published.^17^

### 2.2 Study site

The study was conducted in Muang district, Chiang Rai, the northernmost province of Thailand. The patients with diabetes and/or hypertension were diagnosed and treated by the doctors at the hospital. When they became stable, the patients were referred to the clinics of health centers according to their residential places. All health centers in Thailand are taken care by nurse and public health officers, instead of doctors. There were about 31 health centers in survey site where patients with diabetes and/or hypertension could receive the medications, and we screened all health centers for eligibility criteria. Every cluster had a chance to be allocated to control and intervention arm through the procedure.

### 2.3 Participants

**FIGURE 1:**
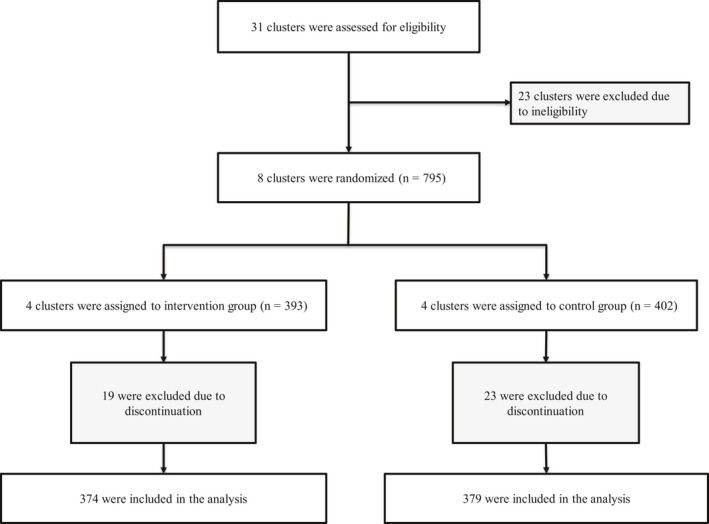
Flow diagram of participants

Figure 1 shows flow diagram of participants. We screened patients who had visited diabetes and/or hypertension clinics of health centers, and enrolled those who met the eligibility criteria which screened participants, the Framingham general CVD risk score (>15%).^17^ Details of the eligibility criteria and exclusion criteria were described in our previous report which analyzed baseline data and enrolled those who met the following eligibility criteria: (a) diabetic and/or hypertensive patients with high CVD risk according to the Framingham general CVD risk score (>15% of the 10 years cardiovascular risk, which were calculated using the information on gender, SBP, total cholesterol [mg/dL], high‐density lipoprotein cholesterol [mg/dL], hypertension medication, cigarette smoker, and diabetes treatment) and (b) patients who were willing to participate in the study. Patients fulfilling the following criteria were excluded: (a) pregnant or trying to become pregnant; (b) age less than 35 years; (c) documented type I diabetes mellitus; (d) undergoing long‐term steroid therapy (more than two weeks); (e) undergoing long‐term nonsteroidal anti‐inflammatory drug (NSAID) therapy (ie, every day for at least one year); (f) cancer; (g) known secondary hypertension such as primary aldosteronism, Cushing's syndrome, or pheochromocytoma; (h) severe chronic pulmonary diseases requiring home oxygen therapy; (i) chronic renal disease (creatinine ≥2.0 mg/dL); (j) congestive heart failure; and (k) a known diagnosis of CVD.^17^, ^18^ Informed consent was obtained from all participants.

### 2.4 Clusters

The units of randomization were health centers. A statistician who did not know about study and intervention was asked to produce random sequenced numbers using Stata software, version 11.^17^ Then, eligible clusters were randomly allocated to the intervention and control groups by simple randomization.^17^

### 2.5 Selecting clusters and randomization

Eligibility of cluster was set in order to prevent an empty cluster as that any health center with a total of fewer than 200 patients attending the hypertension and diabetes clinic was not included. Within each cluster, a total of 100 eligible participants were recruited.^17^

We selected 8 of 31 health centers, applying eligibility criteria for a cluster. The unit of randomization was a cluster, which was a health center. Cluster size was 100, and cluster number was eight. Eligible eight clusters were randomized to four clusters in intervention and four clusters in control arm applying simple randomization. On the launching day of the study, we invited cluster representatives and randomization procedure was conducted. Before randomization, researchers and cluster representatives could not know in which arm each cluster will be located.

After the randomization, clusters were informed about the details of the study procedure, separately for intervention clusters and control clusters, in order to minimize the contamination through second‐hand education message between participants of two arms.

Furthermore, we recognized that we got the control arms and intervention arms, coincidently situating at the opposite side of the motor expressway in the study after random allocation,^17^ and people residing in such opposite places did not cross the road to access health care at primary care units.^17^

### 2.6 Recruitment within a cluster

Among all eligible cases in each cluster of primary care unit, eligible 100 participants were able to be randomly recruited. The cluster representatives knew their allocated arm after random allocation, and the study procedure of only their allocated arm was explained to them. Individual participants were given information pertaining to the study group in their allocated cluster only after enrollment.

### 2.7 Baseline characteristics

Baseline data have been reported previously.^18^ We interviewed participants to collect data on their baseline characteristics including age, gender, medical history (hypertension, dyslipidemia, diabetes mellitus, CVD, cerebrovascular disease, and kidney disease), and family medical history (hypertension, diabetes mellitus, CVD, and cerebrovascular disease). We also inquired about lifestyle characteristics listed in Breslow's 7 health practices and defined the components of a healthy lifestyle, as indicated in parentheses as follows: alcohol consumption (<3 days/week), smoking behavior (current nonsmoker), exercise frequency (≥2 times/week), body mass index (BMI; 18.5‐24.9), sleep hours (6‐9), breakfast consumption habits (every morning), and snacking between meals (no).^19^, ^20^ A healthy lifestyle was defined as having at least 6 healthy practices.

Awareness and motivation related to salt intake were assessed by a self‐administered questionnaire, and detail was already reported as baseline data analysis.^18^ We collected BMI, systolic blood pressure (SBP), diastolic blood pressure (DBP), total cholesterol (mmol/L; TC), high‐density lipoprotein cholesterol (mmol/L; HDL‐C), triglycerides (mmol/L; TG), low‐density lipoprotein cholesterol (mmol/L; LDL‐C) which was estimated using the Friedewald equation ([TC] − [HDL‐C] − [TG/5]),^21^ and glycosylated hemoglobin A1c (National Glycohemoglobin Standard Program [NGSP]).

Daily salt intake, which was the primary outcome, was estimated by A KME‐03 salinity checker (Kono ME Institute, Kanagawa, Japan) using overnight urine samples.^18^, ^22^ Detail of the method of data collection was already reported as baseline data analysis.^17^ We instructed participants to store overnight urine appropriately. The participants then brought the collected urine to the health centers, and staff members measured the estimated 24 hours urinary salt excretion. This procedure was performed for three successive nights, and the average measurement was used as the estimated daily salt intake.

### 2.8 Sample size calculation

Detail of sample size calculation was already described in our previous report.^17^ For a conventional randomized control trial, a total of 240 participants, randomizing 120 into each arm, will have enough power (90%) to detect the minimal difference of systolic blood pressure of 130 (±20) and 120 (±20) mm Hg, diastolic pressure of 90 (±20) and 80 (±20) mm Hg, difference in 24 hours estimated salt intake of 10 (±5) and 8 (±4) g/day, and CVD event rate of 0.15% and 0.08% between the control and intervention groups with a 95% confidence interval.^23^, ^24^ The cluster randomized trial design used in RESIP‐CVD might result in loss of power and reduced efficiency. In this cluster randomized trial, the number of participants in each cluster was the same, and other variable factors such as geography, food tradition, race, and dietary habits were assumed not to be different for delineating the intracluster coefficient. To compensate the design effect, the inflation factor has been calculated by the formula (*Deff = *1 + (*m*−1)*ρ*) where *Deff* is inflation factor for design effect, *m* was the size of each cluster, which was 30, and *ρ* is the intracluster coefficient which is assumed to be 0.07. The sample required for a cluster randomized trial was inflated to 720. With a loss to follow‐up of approximately 10%, we expect that the sample size of 800 patients would yield sufficient power to detect the desired minimal difference in primary and secondary outcomes between the intervention and control groups. All eight clusters of the RESIP‐CVD study will enroll 100 eligible participants.

### 2.9 Intervention

In the intervention group, participants received a health education intervention comprising visualization of the salt content in their typical home‐prepared soup and their estimated daily salt intake, and a small group health education class. The first component of the intervention used a digital handheld pocket PAL‐ES2 salt meter (Atago Co., Ltd), which displays the salt content of their soup, which they brought in from home. This measurement was performed at baseline, 1 month, and 3 months. Dieticians performed the small group education classes at 1 and 3 months after enrollment. These classes lasted two hours and involved participants to reduce daily dietary salt intake by visualizing the salt content of their food as described above and by suggesting ways to prepare palatable meals with low salt content. Only participants in the intervention group were informed of their estimated daily salt intake. Participants in the control group received both routine care services and a brief individual health education session, which included lecture and instruction, not focused on salt reduction.

### 2.10 Outcomes

The primary outcome was estimated daily salt intake, and secondary outcomes were SBP and DBP. Follow‐up was performed at 6 and 12 months. The secondary outcomes of this study were SBP and DBP at 6 and 12 months. SBP and DBP were measured using the automated sphygmomanometer HEM‐907 (Omron Co., Ltd) by well‐trained research nurses and calculated by calculating the mean of two upper arm blood pressure measurements taken for participants who had been seated for at least five minutes.

At each follow‐up, the baseline survey used to assess baseline characteristics was conducted again for each participant. Anthropometric measurements, SBP and DBP measurements, instruction on overnight urine collection, and estimation of daily salt intake from urine were all performed in the same manner as baseline.

### 2.11 Statistical analysis

Data were calculated in individual levels in each group. Results are presented as mean ±  standard deviation (SD) for continuous variables and prevalence (%) for categorical variables. We used two‐sided Student's *t* test for continuous variables and the chi‐square test or Fisher's exact test for categorical variables to compare between the two groups. The McNemar test was used for comparison of categorical variables between baseline and the 6 or 12 month follow‐up within each group. To account for intracluster correlation coefficient (ICC), the mixed‐effects model was conducted.^25^

And then, changes in variables between groups and associated test of effect were estimated by regression adjusting clustering and baseline covariates: body mass index, alcohol consumption (less than 3 days per week), smoking behavior (current nonsmoker), exercise frequency (2 times or more per week), sleep hours (6‐9), breakfast (every morning), snack between meals (no), antihypertensive drug use (yes), antidyslipidemic drug use (yes), antidiabetic drug use (yes), total cholesterol (mg/dL), hemoglobin A1c (%), awareness of seriousness of CVD (yes), and motivation to reduce salt intake (yes).

All significance tests were two‐sided, and *P*‐values less than .05 were considered statistically significant. All data were analyzed using SPSS version 22 (IBM SPSS Inc).

The Japanese Ethics Committee approved the research protocol (No. 2011036), the Thai Ethics Committee approved the research protocol (No. CR0027.102/research/207), and the research protocol was registered in the International Standard Randomized Controlled Trial Number Register (ISRCTN39416277) on January 3, 2012.

## 3 RESULTS

In total, 795 participants were enrolled in the study and 753 completed both follow‐ups (control group: 379; intervention group: 374). Baseline characteristics of participants are shown in Table 1. Mean age was 66.2 (SD 8.6) years in the control group and 66.3 (8.8) years in the intervention group. Proportion of males was 51.7% and 50.8% in the control and intervention groups, respectively. Mean BMI was 24.6 (3.8) in the control group and 24.7 (3.8) in the intervention group. Mean SBP and DBP were 149.8 (19.6) and 75.5 (11.8) mm Hg in the control group, and 149.1 (19.1) and 74.8 (11.6) mm Hg in the intervention group, respectively. As for medications, the proportion of antihypertensive drug use was 84.4% in the control group and 87.7% in the intervention group. In addition, 33.5% and 23.0% used antidyslipidemic drugs, and 44.1% and 42.8% used antidiabetic drugs in the control and intervention groups, respectively. Proportion of participants with a healthy lifestyle was 20.8% in the control group and 25.1% in the intervention group. Mean estimated daily salt intake was 9.8 (2.4) g/day in the control group and 10.0 (2.2) g/day in the intervention group.

**TABLE 1:** Participant characteristics at baseline

	Mean (SD) or N (%)		
	Control group (N = 379)	Intervention group (N = 374)	*P*‐value
Age (years)	66.2 (8.6)	66.3 (8.8)	.82
Male sex	196 (51.7)	190 (50.8)	.80
Anthropometric measurements			
Body mass index	24.7 (3.8)	24.6 (3.8)	.71
Atherosclerotic complications			
CVD	4 (1.1)	6 (1.6)	.51
Cerebrovascular disease	5 (1.3)	6 (1.6)	.74
Kidney disease	3 (0.8)	2 (0.5)	.66
Family history			
CVD	20 (5.3)	7 (1.9)	.01
Cerebrovascular disease	11 (2.9)	8 (2.1)	.50
Hypertension	114 (30.1)	120 (32.1)	.55
Diabetes mellitus	78 (20.6)	80 (21.4)	.79
Hypertension‐related factors			
Systolic blood pressure (mm Hg)	149.8 (19.6)	149.1 (19.1)	.61
Diastolic blood pressure (mm Hg)	75.5 (11.8)	74.8 (11.6)	.45
Heart rate (min)	76.6 (33)	760 (12.0)	.54
Mean arterial pressure (mm Hg)	100.2 (12.4)	99.6 (12.1)	.45
Antihypertensive drug use (yes)	320 (84.4)	328 (87.7)	.20
Lipid‐related items			
Total cholesterol (mg/dL)	178.7 (32.9)	193.7 (37.7)	<.01
High‐density lipoprotein cholesterol (mg/dL)	45.1 (9.8)	41.5 (10.9)	.01
Low‐density lipoprotein cholesterol (mg/dL)	105.2 (30.0)	121.4 (32.5)	<.01
Triglycerides (mg/dL)	195.5 (157.4)	179.4 (101.1)	.31
Antidyslipidemic drug use (yes)	127 (33.5)	86 (23.0)	.03
Diabetes‐related items			
Hemoglobin A1c (%)	6.0 (1.2)	6.3 (1.2)	<.01
Antidiabetic drug use (yes)	167 (44.1)	160 (42.8)	.72
Healthy lifestyle characteristics			
Alcohol consumption (less than 3 days per week)	361 (95.3)	360 (96.3)	.49
Smoking behavior (current nonsmoker)	346 (91.3)	339 (90.6)	.76
Exercise frequency (2 times or more per week)	104 (27.4)	84 (22.5)	.11
Body mass index (18.5‐24.9)	174 (46.9)	189 (51.6)	.20
Sleep hours (6‐9)	244 (64.4)	259 (69.3)	.16
Breakfast (every morning)	361 (95.3)	359 (96.0)	.62
Snack between meals (no)	197 (52.0)	196 (52.4)	.91
Total number of healthy lifestyle items	4.7 (1.0)	4.8 (1.1)	.46
Proportion of participants with 6 or 7 total number of healthy lifestyle items	77 (20.8)	95 (25.1)	.16
Estimated daily salt intake (g/day)	9.8 (2.4)	10.0 (2.2)	.26
Awareness related to salt intake (yes)			
Awareness of CVD risk factors	136 (35.9)	144 (38.5)	.46
Awareness of seriousness of CVD	257 (67.8)	281 (75.1)	.03
Awareness of high salt intake	302 (79.7)	308 (82.4)	.35
Motivation to reduce salt intake	209 (55.1)	249 (66.6)	<.01

Note

Two‐sided Student's *t* test for continuous variables and the chi‐square test or Fisher's exact test for categorical variables was used to compare between the two groups.

Abbreviations: CVD, cardiovascular disease; SD, standard deviation.

Awareness of CVD risk factors and high salt intake were not statistically different between the two groups at baseline. In contrast, awareness of the seriousness of CVD and motivation to reduce salt intake were significantly higher in the intervention group compared to the control group at baseline (67.8% vs 75.1%, 55.1% vs 66.6%, respectively).

**FIGURE 2:**
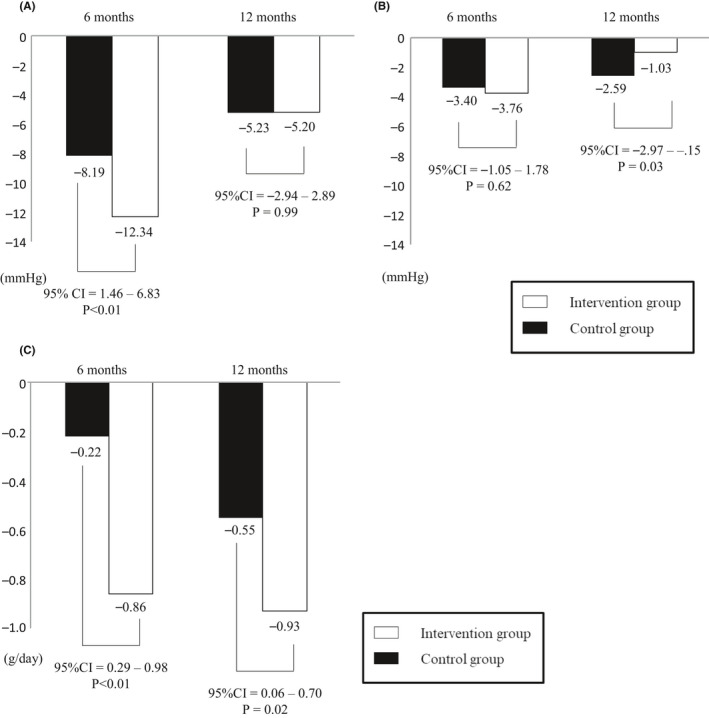
(A) Change in systolic blood pressure. (B) Change in diastolic blood pressure. (C) Change in estimated daily salt intake

Changes in SBP, DBP, and estimated salt intake from baseline to each follow‐up are shown in Figure 2. Decrease in SBP at 6 months was significantly larger in the intervention group compared to the control group (−12.34 vs −8.19 mm Hg, *P* < .01, 95% confidence interval [CI] = 1.46 ‐ 6.83), while there was no significant difference at 12 months (−5.20 vs −5.23 mm Hg. *P* = .99, 95% CI = −2.94 to 2.89) (Figure 2A). Although decrease in DBP was not statistically different between the groups at 6 months (−3.76 vs −3.40 mm Hg, *P* = .62, 95% CI = −1.05 to 1.78), change of the control group was significantly larger than the intervention group at 12 months (−1.03 vs −2.59 mm Hg, *P* = .03, 95% CI = −2.97 to −0.15) (Figure 2B). As for estimated daily salt intake, although the change was significantly larger in the intervention group compared to the control group at both follow‐ups, the difference became smaller at 12 months compared to 6 months (−0.86 vs −0.22 g/day, *P* < .01, 95% CI = 0.29‐0.98 and −0.93 vs −0.55 g/day, *P* = .02, 95% CI = 0.06‐0.70 at 6 and 12 months, respectively) (Figure 2C).

Table 2 shows changes in awareness related to salt intake at baseline and at 12 months. At the 12 month follow‐up, awareness of CVD risk factors and seriousness, awareness of high salt intake, and motivation to reduce salt intake were significantly increased in both groups (*P* < .01). As for the mixed‐effects model, there were significant difference in SBP and estimated salt intake after adjusting covariates at 1st follow‐up (6 months) (adjusted differences between groups [95% CI]; −7.55 [−5.61 to −9.49] mm Hg *P* < .01, −0.66 [−0.40 to −0.92] g/day *P* = .03). However, these differences were not observed at 2nd follow‐up (12 months) (adjusted differences between groups [95% CI]; −1.83 [0.34 to −4.00] mm Hg *P* = .48, −0.42 [−0.17 to −0.67] g/day *P* = .16). There were no differences in DBP in both follow‐ups (Table 3).

**TABLE 2:** Change in awareness related to salt intake

	Control group (N = 379)			Intervention group (N = 374)		
	Number (%)			Number (%)		
	Baseline	12 months	*P*‐value	Baseline	12 months	*P*‐value
Awareness of CVD risk factors	257 (67.8)	292 (77.0)	<.01	281 (75.1)	250 (66.8)	<.01
Awareness of seriousness of CVD	136 (35.9)	183 (48.3)	<.01	144 (38.5)	189 (50.5)	.01
Awareness of high salt intake	302 (79.7)	248 (65.4)	<.01	308 (82.4)	250 (66.8)	<.01
Motivation to reduce salt intake	209 (55.1)	301 (79.4)	<.01	249 (66.6)	357 (95.5)	<.01

Note

The McNemar test was used for comparison of categorical variables between baseline and the 6 or 12 month follow‐up within each group.

Abbreviation: CVD, cardiovascular disease.

**TABLE 3:** Changes in SBP, DBP, and estimated salt intake relative to baseline and regression—estimated differences in change between groups

	Intervention	Control	Available cases analysis			
	Mean (SD)		Adjusted differences between groups (95% CI)	*P*‐value	Effect size	ICC
Change in SBP						
1st follow‐up (6 months)	−12.34 (18.65)	−8.19 (18.83)	−7.55 (−5.61 to −9.49)	<.01	0.22	0.027
2nd follow‐up (12 months)	−5.21 (19.51)	−5.23 (21.20)	−1.83 (0.34 to −4.00)	.48	0.00	0.005
Change in DBP						
1st follow‐up (6 months)	−3.76 (10.43)	−3.40 (9.31)	−1.11 (0.14 to −2.37)	.45	0.04	0.010
2nd follow‐up (12 months)	−1.03 (9.68)	−2.59 (10.04)	1.00 (2.19 to −0.19)	.48	0.16	‐0.010
Change in estimated salt intake						
1st follow‐up (6 months)	−0.66 (2.29)	−0.222 (2.54)	−0.66 (−0.40 to −0.92)	.03	0.26	0.123
2nd follow‐up (12 months)	−0.932 (2.18)	−0.55 (2.29)	−0.42 (−0.17 to −0.67)	.16	0.17	0.086

Note

Changes in variables between groups and associated test of effect estimated regression adjusting clustering and baseline covariates: body mass index, alcohol consumption (less than 3 days per week), smoking behavior (current nonsmoker), exercise frequency (2 times or more per week), sleep hours (6‐9), breakfast (every morning), snack between meals (no), antihypertensive drug use (yes), antidyslipidemic drug use (yes), antidiabetic drug use (yes), total cholesterol (mg/dL), hemoglobin A1c (%), awareness of seriousness of CVD (yes), and motivation to reduce salt intake (yes).

Abbreviations: DBP, diastolic blood pressure; ICC, intracluster correlation coefficient; SBP, systolic blood pressure; SD, standard deviation.

## 4 DISCUSSION

To the best of our knowledge, this RCT was the first trial to assess the effectiveness of a dietary intervention involving cooking instruction and visualization of salt content of food by a digital handheld pocket salt meter in Thailand.

Our results showed that SBP was significantly improved in the intervention group compared to the control group at 6 months, while the significance was not observed at 12 months, suggesting that the intervention may have had a short‐term effect. Several previous trials have indicated the effectiveness of salt intake reduction interventions in lowering BP. An experimental intervention study using a specific diet (emphasizing fruits, vegetables, and low‐fat dairy foods; including whole grains, poultry, fish, and nuts; and low in fats, red meat, sweets, and sugar‐containing beverages) coupled with reduced sodium intake (50, 100, and 150 mmol/d at 2100 kcal) reported that the diet and reduced sodium intake were each associated with significant decreases in BP and that these two factors combined produced the greatest reductions.^13^ The PREMIER trial, which was a multicenter, randomized controlled trial of 810 participants with high‐normal hypertension, revealed that mean SBP declined by 3.7 mm Hg for participants in the established group (consisting of weight loss, increased physical activity, and reduced sodium and alcohol intake) (*P* < .001) and 4.3 mm Hg for those in the established plus DASH (Dietary Approaches to Stop Hypertension) diet group (*P* < .001).^26^ The results of these studies, as well as ours, suggest that a dietary intervention may significantly improve BP in both the general and high‐risk populations.

In addition to BP, estimated salt intake was significantly reduced in the intervention group compared to the control group in our study at first follow‐up. Salt intake reduction is often practically difficult, because of the frequent use of packaged foods, which may have a high salt content. Our intervention program contained cooking instruction using a digital handheld pocket PAL‐ES2 salt meter to display the salt content of their daily food. Several previous studies have reported the efficacy of cooking instruction in dietary modification. A Japanese intervention study of 71 men reported a greater decrease in urinary sodium‐to‐potassium excretion ratio, suggesting lower dietary salt intake, in the intervention group, who received cooking instructions emphasizing fruits and vegetables and self‐monitoring of diet, compared to the control group (net difference 0.6, *P* = .029).^27^ In addition, both SBP and DBP (mm Hg) decreased in the intervention group (149.0‐143.0, *P* = .073; 93.0‐87.0, *P* = .002), but not in the control group (145.0‐143.0, *P* = .231; 84.9‐85.3, *P* = .381). Taken together with our results, an intervention including cooking instruction, such as that implemented in this study, may enable individuals to make low‐salt foods themselves and hence achieve reduced salt consumption.

In our study, awareness relating to salt intake improved from baseline to follow‐up in both groups. This may explain why changes in SBP and estimated daily salt intake did not differ significantly at 12 months. A number of studies have examined the relationship between awareness and behavior related to salt intake and actual salt intake. An epidemiological study evaluating 742 Japanese adults reported that responses to simple questions regarding excess salt intake did indeed correspond to excess salt intake.^28^ An observational study of 189 Korean adults assessing the correlation between salt usage behavior assessed by a questionnaire and 24 hours urinary sodium excretion showed that among 15 questions, scores of 3 questions on salt usage behavior were significantly correlated to urinary sodium excretion (*r* = .17‐.19; *P* < .05) and the sum of scores of the 3 questions showed higher correlation coefficients (*r* = .26, *P* < .001).^29^ Moreover, interventions targeting awareness and behavior may be effective in reducing salt intake and BP. A community‐based intervention, which aimed to improve salt‐related knowledge and behavior using a Communication for Behavioral Impact (COMBI) intervention program among 513 Vietnamese participants, reported that mean sodium excretion estimated from spot urines fell significantly, from 8.48 g/d at baseline to 8.05 g/d at follow‐up (*P* = .001). Mean SBP and DBP were also significantly lower following the intervention (−5.93 mm Hg [95% CI, −8.03 to −3.83; *P* < .001] and −4.86 mm Hg [−6.21 to −3.51; *P* < .001], respectively).^30^ In fact, the results showed a positive change in knowledge and behavior related to salt consumption between baseline and follow‐up.^30^ Thus, awareness and behavior (physical activity, weight loss, adherence to drug therapy, and so on) concerning salt intake may correlate with actual salt intake. In our study, visualizing their estimated salt intake may have further motivated participants to reduce salt intake in short term.

Our study has several limitations worth noting. First, selection bias may have been present, and participation was limited to those who lived in one area of Thailand. Further analyses are needed including participants widely from many districts of Thailand. Second, we registered only participants with higher cardiovascular risks in the present study, and it is required to assess participants with low cardiovascular risks in future analysis. Third, the automated device used to estimate 24 hours salt intake based on overnight urine was validated in Japanese individuals, but not in other ethnicities. In addition, validity and reliability of a digital handheld pocket salt meter were not fully estimated. To assess the validity and reliability of a digital handheld pocket salt meter in other ethnicities, additional study is needed. Fourth, we could not collect the detail of medication including diuretics and could not consider the effect of diuretics on urinary sodium excretion, and validated assessment considering diuretics is required. Fifth, we did not collect the detail of other dietary and nutrient information such as potassium and protein intake, and it is possible that our results will be modified after considering the factors. It should be considered in the future. Sixth, the statistical analysis of the effect of intervention in RCTs should follow the intention‐to‐treat principle. However, 19 of 393 in intervention group and 23 of 403 in control group could not continue the study, and we could not measure the outcomes (completion rate; 95.2% in intervention group and 94.3% in control group). Seventh, the present study was not conducted according to the double‐blinded protocol. Therefore, participants were aware of in which group they were allocated. It might affect the results. The study, which is considered with blinding, is needed in the future. Eighth, BP was only measured once in each visit, which may lead to overestimates of underlying BP. Repeated measures at the same visit are needed in the future. Ninth, we could not have surveyed detail of the changes of characteristics exactly during follow‐up period. It is possible that some changes might affect the outcomes, and it should be considered in the future.

## 5 CONCLUSION

Our trial data revealed the effectiveness of a dietary intervention combining cooking instruction with visualization of SBP and estimated salt intake in short term, while the effectiveness discontinued in long term. This type of dietary intervention targeting salt intake reduction may be required for BP management, and a developed intervention is necessary for long‐term management.

## CONFLICT OF INTEREST

The authors have no conflicts of interest to declare.

## ETHICAL CONSIDERATION

The Ethics Committee of Juntendo University approved the research protocol (No. 2011036), the Ethics Committee of Chiang Rai Regional Hospital approved the research protocol (No. CR0027.102/research/207), and the research protocol was registered in the International Standard Randomized Controlled Trial Number Register (ISRCTN39416277) on January 3, 2012.

## Supporting information

Supplementary Material
